# Mechanisms of Action for Antimicrobial Peptides With Antibacterial and Antibiofilm Functions

**DOI:** 10.3389/fmicb.2019.02866

**Published:** 2019-12-12

**Authors:** Nigare Raheem, Suzana K. Straus

**Affiliations:** Department of Chemistry, The University of British Columbia, Vancouver, BC, Canada

**Keywords:** antimicrobial peptide, host defense peptide, mechanism of action, biological assays, biophysical methods, antimicrobial resistance

## Abstract

The antibiotic crisis has led to a pressing need for alternatives such as antimicrobial peptides (AMPs). Recent work has shown that these molecules have great potential not only as antimicrobials, but also as antibiofilm agents, immune modulators, anti-cancer agents and anti-inflammatories. A better understanding of the mechanism of action (MOA) of AMPs is an important part of the discovery of more potent and less toxic AMPs. Many models and techniques have been utilized to describe the MOA. This review will examine how biological assays and biophysical methods can be utilized in the context of the specific antibacterial and antibiofilm functions of AMPs.

## Antibiotic Resistance and AMPs

With the problem of increasing antibiotic resistance and shortage of new antibiotics, the need for novel and effective alternatives is essential (Levy and Bonnie, 2004; Hancock and Sahl, 2006; Stanton, 2013; Haney et al., 2019). The emergence and prevalence of multidrug resistant bacterial strains are becoming commonplace (World Health Organization [WHO], 2014). For instance, Gram-positive bacteria such as *Staphylococcus aureus* have been linked to a high proportion of hospital acquired infections (Malanovic and Lohner, 2016). Persistent *S. aureus* colonization occurs in 20% of the population and can cause severe pathogenic infections including sepsis, pneumonia, meningitis and even death (Otto, 2010; Malanovic and Lohner, 2016). Methicillin resistant strains of *S. aureus* (MRSA) are treated with vancomycin administration, however, vancomycin resistant strains have emerged as well (Weigel et al., 2003; Rossi et al., 2014). In addition to the above MRSA-related examples, infections resulting from *Acinetobacter baumannii* for instance, are estimated to be $34k–134k per infection (Nelson et al., 2016). These bacteria are part of the group known as the ESKAPE pathogens (*Enterococcus faecium*, *S. aureus*, *Klebsiella pneumoniae*, *A. baumannii*, *Pseudomonas aeruginosa*, and Enterobacter species) and are the leading cause of nosocomial infections worldwide (Santajit and Indrawattana, 2016). Most of these bacteria are multidrug resistant isolates, making eradication difficult.

Since their discovery in the late 1980s, antimicrobial peptides (AMPs) have been viewed as one of the important solutions to the impending crisis of antimicrobial resistance (AMR) (Hancock, 2001; Zasloff, 2002; Straus and Hancock, 2006; Pan et al., 2007; Haney et al., 2012; Haney and Hancock, 2013; Uhlig et al., 2014). AMPs are naturally occurring polypeptide sequences comprised of cationic and hydrophobic amino acids (∼12–50 residues) with direct antibacterial activity. AMPs are often introduced in the literature as “promising alternative to antibiotics,” “potential to address the growing problem of antibiotic resistance,” and “hold promise to be developed as novel antibiotics” (Haney et al., 2019). AMP discovery and development either involves identifying novel peptides from natural sources [e.g., organisms and tissue extracts (Mangoni et al., 2015; Rodríguez-Decuadro et al., 2018; Yang D. et al., 2018; Yang L.L. et al., 2018), excised predicted antimicrobial sequences from larger proteins (Pane et al., 2016; Abdillahi et al., 2018; Pizzo et al., 2018)] or optimizing antibacterial activity by making synthetic variants (Bahar and Ren, 2013; Haney and Hancock, 2013; Zhao et al., 2013; Kim et al., 2014, 2016; Wang et al., 2016; Kumar et al., 2017; Travkova et al., 2017; Akbari et al., 2018; Chen et al., 2018). The aim is generally to find a handful of peptide sequences with broad spectrum antimicrobial activity toward antibiotic resistant pathogens. However, many of these peptides have more than one function: many AMPs have been shown to possess immunomodulatory, anti-cancer and antibiofilm functions in addition to their antimicrobial properties (Powers and Hancock, 2003; Chan et al., 2006; Hancock and Sahl, 2006; Pletzer and Hancock, 2016; Andrea et al., 2018; Kumar et al., 2019). Indeed, this ability to act on multiple fronts is what makes AMPs attractive (Hancock and Sahl, 2006; Haney et al., 2019) and has led to them being referred to as host defense peptides (HDPs).

## Host Defense Peptides

**FIGURE 1:**
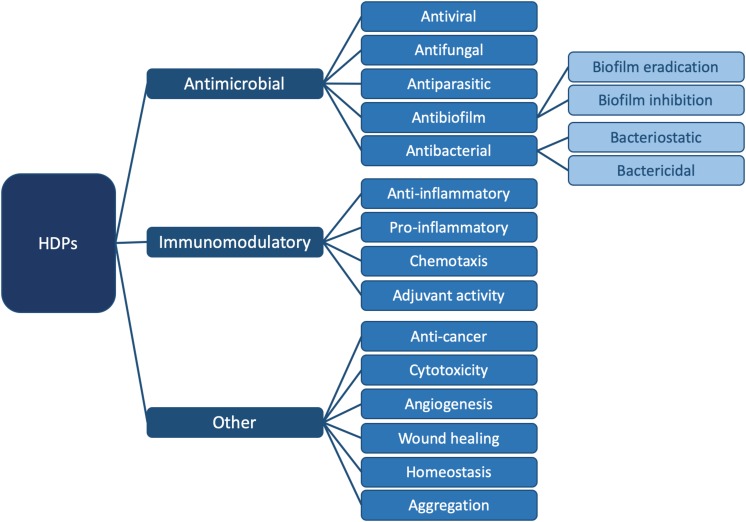
Summary of known functions for HDPs. In this review, we focus on the antibacterial (Figure 2) and antibiofilm (Figure 3) functions in particular. For specific examples of HDPs displaying antiviral, antifungal, antiparasitic, immunomodulatory, and other functions, please consult (Haney et al., 2019).

In order to emphasize the multifaceted nature of AMPs, the term “HDP” was coined (Bowdish et al., 2005a; Hancock and Sahl, 2006; Nijnik and Hancock, 2009; Takahashi et al., 2010). HDPs are involved in a breadth of biological processes due to their versatility (Bowdish et al., 2005b; Ulm et al., 2012); they are ubiquitous in nature and are part of the innate immune defense system of almost all life forms (Hancock, 2005; Jenssen et al., 2006). They can modulate the immune response, demonstrate anti-cancer activity and inhibit or eradicate biofilms. They can kill bacteria directly, by either targeting a broad spectrum of bacteria, or by being selective for Gram-positive or Gram-negative bacteria. Finally, HDPs are also active against pathogenic species such as viruses, fungi and parasites (Hancock and Falla, 1996; Hancock and Sahl, 2006; Travkova et al., 2017). Figure 1 summarizes all currently known functions of these diverse biomolecules.

Although an increasing number of studies examine more than just the AMP function (i.e., direct killing of bacteria, Figure 1) (e.g., Kumar et al., 2019), much remains to be understood about how HDPs work. In particular, it remains to be determined whether the multiple functions of HDPs are independent from one another or whether some functions have commonalities. By understanding how amino acid sequence modulates more than just antibacterial activity, the true therapeutic potential of HDPs could be harnessed. As discussed in detail in Haney et al. (2019), recognizing the emerging roles and activity landscape of HDPs will lead to the development of drugs with effectiveness against infectious diseases as well as inflammatory conditions. For nearly 40 years, HDPs have been championed as one of the important tools to combat AMR. Unfortunately, this promise remains to be fulfilled [e.g., resistance mechanisms to AMPs have been reported as discussed in Haney et al. (2019)]. In fact, other than polymyxin B and gramicidin S being used as topical agents, only a few peptides have entered clinical trials (Hancock and Sahl, 2006; Haney and Hancock, 2013; Haney et al., 2019). Nevertheless, the multi-faceted nature of HDPs and their ability to influence a wide range of biological processes warrants further study. A key to moving the field forward is to understand the mechanistic details underpinning the multiple functions of HDPs. In this review, we examine how biological assays and biophysical tools can shed light into the mechanism of action (MOA) of HDPs, important information that can potentially be leveraged to more fully realize the therapeutic promise of HDPs (Haney et al., 2019).

## Motivation for Studying HDP Mechanism of Action

Ever since AMPs were first discovered four decades ago, scientists have tried to relate amino acid sequence to antibacterial activity, i.e., to derive design rules to yield “better” peptides. The typical approach is to substitute amino acids in the sequence in order to manipulate cationic charge and hydrophobicity. This generally results in a small library of ∼5–10 peptides, which are tested for antimicrobial activity. In most published examples, some derivatives exhibit moderately enhanced antimicrobial potency relative to the parent sequence (Fjell et al., 2012; Haney et al., 2012; Kim et al., 2014, 2016; Kumar et al., 2018), or perhaps lower toxicity, and perhaps ∼1–4 of these peptides are studied further to determine their MOA. So although databases with large numbers of sequences exist, e.g., the AMP Database with over 3000 entries^1^ (Wang et al., 2016), DRAMP with over 17000 entries (Fan et al., 2016), DBAASP^2^ (Pirtskhalava et al., 2016) and others, the MOA for only a small proportion of these peptides is known. The primary reason for this, of course, is that determining MOA can be labor intensive, as it requires multiple experiments. In addition, the MOA is usually characterized for the antibacterial function of the peptide and not necessarily for its other functions.

**FIGURE 2:**
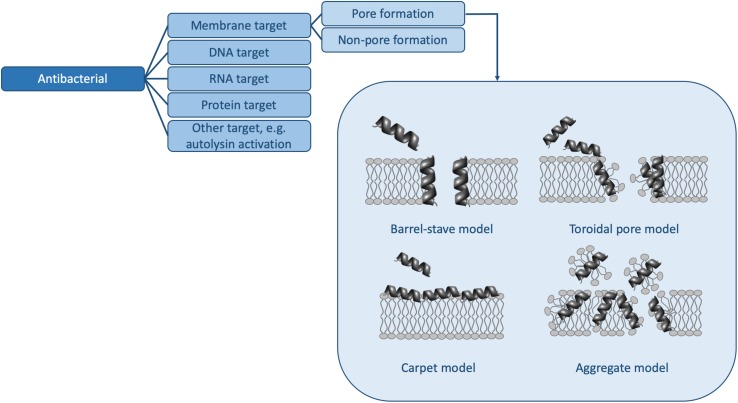
Mechanisms of action for antibacterial HDPs. The pore forming mechanisms, which have been characterized extensively in many papers (e.g., reviewed in Kumar et al., 2018), are shown in more detail. Table 1 contains detailed examples of HDPs which function via each of the listed MOAs.

**FIGURE 3:**
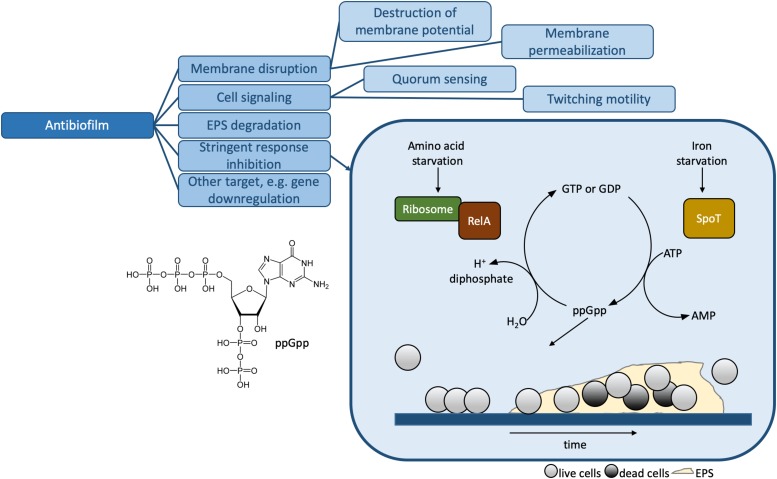
Mechanisms of action for antibiofilm HDPs. The stringent response inhibition mechanism involving ppGpp is shown in more detail. The presence of the alarmone leads to the formation of biofilms, illustrated at the bottom of the box. The biofilm consists of live cells (light gray circles), dead cells (dark gray circles) and the extracellular polymer substance or EPS. Table 1 contains detailed examples of HDPs which function via each of the listed MOAs.

However, in order to truly understand the HDP activity landscape (Haney et al., 2019), more information is required: i.e., we need to generate a large number of sequences [e.g., using SPOT synthesis (Winkler et al., 2009; Haney et al., 2015)], to collect activity data for each of these peptides, and to determine MOAs for representative members. In the following, we examine some of the methods available to us to determine activity and MOA and discuss how future studies (and databases) may contain sufficient information to strengthen HDP design rules. Because HDPs have many functions and because there are many methods to determine similar parameters, the sections below do not represent an exhaustive list of assays available, but rather some of the more common ones. Moreover, we will limit ourselves to the antibacterial (Figure 2 and Table 1) and antibiofilm (Figure 3 and Table 1) MOAs of HDPs.

**TABLE 1:** Summary of antibacterial and antibiofilm MOAs, assays and techniques used to characterize these MOAs and representative HDPs.

	**Mechanism of action**	**Assays/Techniques**	**Representative HDPs (references)**
Antibacterial	Membrane target	MIC; MBC – bactericidal^a^/Membrane depolarization (DiSC_3_5 assay; pyranine leakage); Membrane damage (Sytox Green, PI, calcein leakage, ion leakage, DNA/RNA release, OCD, DSC, NMR, SEM); Cell wall targets (e.g., LPS, lipid II) – NMR, ITC, SPR	**Aurein 2.2** (Cheng et al., 2011; Wenzel et al., 2015); **Magainin** (Matsuzaki et al., 1998); **HNP-1** (de Leeuw et al., 2010); **Cg-Defh1** (Schmitt et al., 2010); **Thanatin** (Sinha et al., 2017); **Esculentin-1a** (Luca et al., 2013); **LL-37** (Zeth and Sancho-Vaello, 2017)
	DNA target	MIC; MBC – bactericidal^a^/Gel electrophoresis	**Buforin II** (Park et al., 1998; Kobayashi et al., 2000, 2004; Xie et al., 2011); **Indolicidin** (Hsu et al., 2005; Marchand et al., 2006; Hale and Hancock, 2007; Hilpert et al., 2010)
	RNA target	MIC; MBC – generally bacteriostatic^a^/Gel electrophoresis	**Attacin** (Carlsson et al., 1991)
	Protein target	MIC; MBC – generally bacteriostatic^a^/Co-precipitation; fluorescence	**Bac71-35** (Mardirossian et al., 2014); **Api137** (Krizsan et al., 2015); **Tur1A** (Mardirossian et al., 2018)
	Other target	MIC; MBC e.g., autolysin release	**Mel4** (Yasir et al., 2019)
Antibiofilm	Membrane disruption	MBIC; MBEC/Membrane depolarization (DiBAC4(3) assay); Membrane damage (Sytox Green, PI, Syto-9, ATP release)	**Esculentin-1a** (Luca et al., 2013); **Nisin A, Lacticin Q, Nukacin ISK-1** (Okuda et al., 2013)
	Cell signaling	MBIC; MBEC	**LL-37 and Indolicidin** (Overhage et al., 2008); **1037** (de la Fuente-Núñez et al., 2012)
	EPS degradation	MBIC; MBEC	**Hepcidin-20** (Batoni et al., 2016)
	Stringent response inhibition	MBIC; MBEC/Co-precipitation; ^31^P NMR	**1018** (de la Fuente-Núñez et al., 2014)
	Other target	MBIC; MBEC e.g., gene down-regulation/targeting	**Nal-P-113** (Wang et al., 2017); **human β-defensin 3** (Zhu et al., 2013)

*^*a*^Based on (Finberg et al., 2004).*

## Biological Assays

In this review, we will use the term “biological” to refer to methods which make use of bacteria (as opposed to individual components, i.e., model lipid membranes, DNA, etc.), in either planktonic (free swimming) or biofilm form. Biofilms are microorganism aggregates associated with surfaces, surrounded by an extracellular polymer substance (EPS; Figure 3) consisting of polysaccharides, extracellular DNA, proteins, lipids, and water (de la Fuente-Núñez et al., 2014, 2016; Flemming et al., 2016). Biofilm formation is an adaptation of planktonic bacteria to environmental stress factors such as antibiotics, host immune system, starvation, and others (de la Fuente-Núñez et al., 2016; Pletzer and Hancock, 2016). Planktonic bacteria respond to stress factors by triggering the stringent response, a conserved mechanism in both Gram-positive and Gram-negative bacteria mediated through the synthesis of signaling nucleotide (p)ppGpp (de la Fuente-Núñez et al., 2014; Pletzer and Hancock, 2016; Figure 3). The EPS provides enhanced AMR to biofilms compared to planktonic bacteria (Flemming et al., 2016).

In addition, we will limit ourselves to *in vitro* assays. For excellent articles on the use of *in vivo* models to characterize HDP antibacterial and antibiofilm activity, the reader is invited to consult (Haney and Hancock, 2013; Pletzer et al., 2017a, b, 2018) and references therein.

### Biological Assays to Determine Activity

#### Minimum Inhibitory Concentration

Minimum inhibitory concentration (MIC) is the lowest concentration (typically reported in μg/mL or μM) required of an antimicrobial agent to prevent the growth of planktonic bacteria (Wiegand et al., 2008). For HDPs, values are typically in the 1–32 μg/mL range, as compared to <1 μg/mL typically observed for antibiotics. The higher MIC values observed for HDPs are likely due to the fact that the presence of rich media or high salt affects cationic peptide activity (Mahlapuu et al., 2016; Starr et al., 2016; Haney et al., 2019). MIC is not only a useful parameter for assessing the therapeutic potential of new antibiotic candidates, but also permits the classification of bacteria as being clinically susceptible or resistant to the tested antibiotic (Wiegand et al., 2008). *In vitro* techniques such as agar dilution or broth dilution are commonly used for determining the MIC of antimicrobials (Wiegand et al., 2008).

In the agar dilution method, solutions containing a certain number of bacterial cells are spotted onto agar plates that contain different antibiotic concentrations. The observation of bacterial colonies on these plates after incubation indicates bacterial growth. The broth dilution method involves the use of a liquid growth medium containing a certain number of bacterial cells for inoculation and to which an increasing concentration of the antibiotic is added. The growth of bacteria is indicated by the presence of turbidity or sedimentation after a period of incubation. For both methods, the lowest concentration of antimicrobial agent needed to prevent the visible growth of bacteria is defined as MIC (Wiegand et al., 2008).

Other much less common methods to determine MIC include the agar diffusion method and the antimicrobial gradient method (Etest) (Bonev et al., 2008; Wiegand et al., 2008; Balouiri et al., 2016). In the agar diffusion method, an agar plate is inoculated with the test bacterial strain. Then filter paper discs, wells, strips or cups containing known concentrations of the test antimicrobial agent are placed on or punched into the agar plate (Bonev et al., 2008). The diffusion of an antibiotic into the agar medium inhibits bacterial growth. The MIC value can be determined from the relationship between the size of growth inhibition zones and the antibiotic concentration (Bonev et al., 2008). A disadvantage of this technique is that the obtained MIC value is often not accurate as the test antibiotic may not diffuse freely in the solid medium and could lead to partial inactivation (Bonev et al., 2008). The Etest method uses a strip containing an increasing concentration of the test antimicrobial agent, which is placed onto the agar plate already inoculated with the test microorganism. After incubation, the MIC value is obtained from the intersection of the growth inhibition zone with the test strip (Balouiri et al., 2016). Studies have shown good correlation of MIC results obtained from Etest and broth/agar dilution method (Balouiri et al., 2016). However, the disadvantages of this method include the cost for testing numerous antimicrobial agents, as well as the limited antibiotic concentration range set by the manufacturer (Wiegand et al., 2008). Since both these approaches have important shortcomings, they have only been used in a limited number of studies and are no longer common practice (Jevitt et al., 2006; Galani et al., 2008).

The methods described above can be readily adapted to screen a number of peptides, with the broth dilution method being commonly used to screen large peptide libraries (Jenssen et al., 2007; Haney and Hancock, 2013; Kumar et al., 2017) obtained from SPOT-synthesis (Winkler et al., 2009). In this case, 96-well plates are typically used and the turbidity is measured by recording the optical density (OD) using a plate reader. In the future, it is conceivable that microfluidic devices may even be used (Lee et al., 2017).

#### Minimum Biofilm Inhibitory Concentration

The techniques presented in the previous section enable the assessment of the antimicrobial activity against planktonic bacteria. Since bacteria can also exist as biofilms, assays specific to bacteria in this state are required. The minimum biofilm inhibitory concentration (MBIC) is the lowest concentration of antimicrobial agent required to inhibit biofilm formation. An HDP is considered to be antibiofilm if the MBIC is below the MIC, with a distinct structure activity relationship compared to the direct killing antimicrobial activity (de la Fuente-Núñez et al., 2016). Different susceptibility tests can be done to determine the MBIC, including the use of an abiotic solid surface as well as the flow cell technique (Pitts et al., 2003; Overhage et al., 2008; de la Fuente-Núñez et al., 2012).

For instance, 96-well microtiter plates with sterile growth media are first inoculated with bacteria to allow growth, and then different concentrations of antimicrobial agents are added to observe biofilm prevention (Macià et al., 2014; Gordya et al., 2017). After a period of incubation, the spent culture fluid and planktonic bacteria are removed prior to cell staining with crystal violet dye, which is then dissolved in ethanol or glacial acetic acid (Macià et al., 2014). Pipetting helps detach the biofilm in each well and the OD at 570 nm can be measured using a microplate reader for biomass assessment (Macià et al., 2014; Cruz et al., 2018). Since crystal violet stains both dead and living cells, alternative dyes have been used. For example, the metabolic reducing dye MTT (3-(4,5-dimethyl-2-thiazolyl)-2,5-diphenyltetrazolium bromide) can be utilized (Segev-Zarko et al., 2015). Viable cells reduce the MTT dye and lead to color formation: the OD at 570 nm can hence be correlated to the number of living cells per well. The lack of visible color from an MTT-assay indicates the MBIC value of the antimicrobial agent (Nair et al., 2016). Alternatively, biofilms can be incubated with the blue phenoxazine dye resazurin, which is converted to resorufin in the presence of viable cells. Measuring resorufin fluorescence using a plate spectrophotometer indicates the metabolic activity of biofilm cells (Macià et al., 2014). Biofilm cells can be quantified afterward by suspending the biofilm in each well, followed by the agar plate count method (Cruz et al., 2018).

In addition to being able to distinguish between dead and living cells, assessment of biofilm inhibition requires that a clear distinction be made between planktonic and biofilm bacteria. To test whether the antimicrobial agent acts on a uniform biofilm, a so-called Calgary biofilm device (CBD) can be used (Ceri et al., 2001). This device consists of an upper lid containing 96 polystyrene pegs, which can be fitted in the channels of the bottom component and the wells of a 96-well microtiter plate. The bottom component allows consistent medium flow to all pegs to ensure even biofilm deposition (Ceri et al., 2001). Afterward, the peg lid is rinsed and transferred to a 96-well plate with different concentrations of antimicrobial agent in each well and then incubated overnight. The peg lid is rinsed again and placed into a second 96-well plate to transfer the biofilms from each peg into the wells containing growth medium, which is achieved by sonication or light centrifugation (Ceri et al., 2001; Macià et al., 2014). Viability of biofilm cells can be assessed by measuring turbidity at 650 nm (Ceri et al., 2001). For MBIC determination, OD_650_ is measured both before and after plate incubation at 37°C for 6 h and then compared to the positive control wells. The concentration of antimicrobial agent that leads to an OD difference which is ≤10% of the average of two positive control wells is defined as the MBIC value (Macià et al., 2014). For a schematic of the CBD device, the reader is referred to excellent papers describing this and other approaches (*vide infra*) (Gu et al., 2015; Azeredo et al., 2017; Shi et al., 2019).

The above-mentioned techniques are simple and reproducible, but do not fully capture *in vivo* conditions (Macià et al., 2014). The flow cell system is a more sophisticated method for evaluating antibiofilm activity (de la Fuente-Núñez et al., 2012; Quilès et al., 2016). In this technique, biofilms are grown in flow cell chambers in the presence of HDPs at specific concentrations and imaged non-destructively using confocal laser scanning microscopy (CLSM) or Attenuated Total Reflectance–Fourier Transform Infrared (ATR–FTIR) spectroscopy (Quilès et al., 2016). Bacteria are fluorescently tagged for CLSM analysis by using green fluorescent protein, cyan fluorescent protein, or yellow fluorescent protein (Macià et al., 2014). In addition, propidium iodide (PI) can be used to stain dead cells for studying the bactericidal ability of an antibiofilm agent (Macià et al., 2014). Dispersed bacteria cell counts can also be measured by plating the output flow on LB agar plates (de la Fuente-Núñez et al., 2014). For the ATR–FTIR experiment, no labeling is required: instead the characteristic IR signals arising from the proteins, nucleic acids and polysaccharides in the biofilm are tracked as a function of time (Quilès et al., 2016). From the flow cell data, both biofilm growth inhibition and eradication of existing biofilms can be studied (de la Fuente-Núñez et al., 2012, 2014). Finally, instead of flow cell chambers, a suspended substratum reactor, also known as the CDC biofilm reactor, can be utilized. This reactor contains coupons suspended from the coupon holder lid into the growth medium-containing reactor. Bacterial incubation allows biofilm growth on the coupons. The presence of antimicrobial agents in the fluid phase allows complete exposure of all coupons. The coupons can be removed at different time intervals during the experiment and quantification can be achieved afterward by plate counting. The biofilm structure can be examined by CLSM, similar to the flow cell method (Macià et al., 2014).

#### Minimum Biofilm Eradication Concentration

Minimum biofilm eradication concentration (MBEC) is the minimum concentration of an antimicrobial agent required to eliminate pre-formed biofilms. Similar to MBIC determination, the methods outlined above can be used. Generally, MBECs are larger than MBICs, as eradication of biofilms is more difficult than inhibition (Cruz et al., 2018). Bacterial biofilms need to be formed before the addition of antimicrobial agents to test eradication (Cruz et al., 2018). Recently, an improved method which relies on the use of a dissolvable bead has helped to improve the reliability and robustness of MBEC determinations (Dall et al., 2017). The alginate beads result in a more homogeneous biofilm and a more homogeneous exposure of the antimicrobial, resulting in a more responsive assay than surface-based methods.

### Biological Assays to Determine MOA

Once the concentration required to kill planktonic bacteria, to inhibit or eradicate biofilms has been determined, it is useful to assess how these processes occur. For instance, the MIC does not allow a differentiation between the bacteriostatic and bactericidal activities of an HDP. Furthermore, an HDP may be bactericidal and eradicate biofilms (Dall et al., 2018; Swedan et al., 2019). Bacteriostatic antibiotics act by fully or partially inhibiting bacterial growth (Wiegand et al., 2008); however, growth will resume after removal of the antibiotic. In contrast, bactericidal antibiotics cause cell death (Schwalbe et al., 2007). Several studies have suggested that in general terms, bacteriostatic and bactericidal activities can be linked to MOA (Finberg et al., 2004; Woodburn et al., 2019; Yasir et al., 2019), though exceptions exist (Finberg et al., 2004). In the following, we present methods to determine the bactericidal activity of HDPs, as well as methods to look at membrane perturbation. As before, these methods rely on the use of bacteria.

#### Minimal Bactericidal Concentration

The minimum bactericidal concentration (MBC) is defined as the minimum concentration for an antimicrobial agent to kill 99.9% of a bacterial inoculum (Schwalbe et al., 2007). The assay is generally performed by broth macro- or micro-dilution of the bacterial sub-culture. An appropriate amount of antimicrobial agent and the bacteria are mixed together for a time (e.g., 1 h). This mixture is then plated on non-selective agar plates and incubated for ca. 24 h. The remaining colony forming units (CFU) are counted to determine the MBC. In contrast to MIC which provides inhibitory activity information, MBC gives information on the bactericidal activity of the test antibacterial (Schwalbe et al., 2007; Balouiri et al., 2016) or antibiofilm (Pulido et al., 2016) agent at a specific time point.

Alternatively, the MBC value can be determined at different time points. This is known as a time-kill assay and allows the determination of the bacterial killing rate, as well as the concentration dependence of the antimicrobial agent (Schwalbe et al., 2007; Luca et al., 2013). Using this approach, bacterial growth is studied in the absence (growth control) and presence of different concentrations of the antimicrobial agent, typically expressed as fractions or multiples of MIC (e.g., 0.25–2 × MIC). Serial dilutions are performed on the aliquot of culture removed at certain time intervals, followed by the agar plate count method to determine viable cells after a period of incubation. The result is usually plotted with the number of surviving cells [typically expressed as log_10_(CFU/mL)] on the ordinate and time (in hours or minutes) on the abscissa. A greater than 3 log_10_-fold decrease in surviving cells corresponds to a 99.9% killing of the initial inoculum, which is indicative of bactericidal activity at the test antimicrobial concentration (Schwalbe et al., 2007). Time-kill assays can also be used to test the synergistic behavior of different antimicrobial agents (Schwalbe et al., 2007).

#### Cytoplasmic Membrane Disruption

The membrane disruptive activity of an HDP can be examined by using a number of dyes. The first dye, 3,3-dipropylthiacarbocyanine (DiSC_3_5) (Sims et al., 1974), can be used to examine membrane depolarization. Alteration of the trans-membrane electric potential is correlated with a change in membrane permeability as well as cell death once past a critical point (Halder et al., 2015). The fluorescent cyanine dye can be taken up into the bacterial cytoplasm depending on the trans-membrane potential; the accumulation of the dye leads to a quenching of its fluorescence (Zhang et al., 2000; Jung et al., 2008; Cheng et al., 2010; Rasul et al., 2010). Upon addition of the antimicrobial agent, the cytoplasmic membrane is permeabilized and depolarized, leading to dye release and fluorescence increase. The increase in fluorescence indicates the extent of membrane potential depolarization, as well as the integrity of the bacterial cells. The viability of cells upon antimicrobial action can be assessed at different time intervals by plate counting to determine the CFU (Zhang et al., 2000; Pulido et al., 2016).

In contrast to Gram-positive bacteria, the presence of outer membrane (OM) in Gram-negative bacteria acts as a barrier to prevent DiSC35 dye from reaching the cytoplasmic membrane. In order to allow dye accumulation within the cytoplasm, several agents can be used to permeabilize the OM while not causing cell death. For instance, the metal ion chelator ethylene diamine tetra-acetic acid (EDTA) can be used to bind divalent ions such as Ca^2+^ and Mg^2+^ that are present in the OM, which then leads to dye uptake into the cytoplasm (Yang S.T. et al., 2006). Polymyxin derivatives such as polymyxin B nonapeptide can perturb the OM of Gram-negative bacteria by binding to lipopolysaccharide (LPS), which leads to increased OM permeability toward hydrophobic antimicrobial agents (Tsubery et al., 2000; Delcour, 2009). In addition, the AMP ceragenin can also increase permeability of the OM, hence increasing the sensitization of Gram-negative bacteria toward hydrophobic antibiotics (Epand et al., 2010).

Other dyes such as Sytox Green or PI can be used to establish whether HDPs damage cytoplasmic membranes and interact with intracellular nucleic acids (Sträuber and Müller, 2010; Davey and Hexley, 2011; Wenzel et al., 2015; Yang Y. et al., 2015; Pulido et al., 2016; Yasir et al., 2019). These dyes penetrate through membranes in which pores are present and can be used to probe differences in pore size and structure. The protocol (Li et al., 2012) typically involves suspending bacterial cells and dispensing them into wells of 96-well plates along with the dye. After a period of incubation, HDP is added and a change in fluorescence is observed. For example, once PI enters cells with damaged membranes (i.e., dead cells), it binds to nucleic acids and leads to an increase in red fluorescence (Davey and Hexley, 2011). Triton X-100 is often used as the control for complete membrane damage. Alternatively, flow cytometry techniques can be used (Orman and Brynildsen, 2013) in a high-throughput fashion and dyes that stain viable cells, e.g., Syto-9, can be used in conjunction with membrane impermeable dyes to obtain a complete picture of the extent of cytoplasmic membrane disruption by an HDP (Yasir et al., 2019).

#### Leakage of Intracellular Components

Membrane disruption can be further characterized by measuring which components leak out of bacterial cells, i.e., ions, ATP, or DNA/RNA. For example, cellular ions, phosphorus, and sulfur can be detected by atomic emission spectroscopy after exposing bacterial cells to HDPs (Wenzel et al., 2014, 2015; Li et al., 2016). For select ions, such as potassium, leakage can also be determined using a selective electrode (Tsutsumi et al., 2012; Wenzel et al., 2012; Hou et al., 2019). Likewise leakage of ATP can be assessed by using an ATP bioluminescence kit and comparing total vs. released amounts (Gottschalk and Thomsen, 2017; Hou et al., 2019; Yasir et al., 2019). This can also be done in the context of biofilms (Okuda et al., 2013). Finally, DNA and RNA can be detected by using the fact that these molecules contain chromophores which can be detected using UV spectrometry (i.e., by measuring at OD_260 nm_) (Yasir et al., 2019). These methods are generally not used in a high-throughput fashion, but are useful for pinpointing details of the MOA.

#### Other Methods

Most of the methods outlined above are used to separate out HDPs which function by membrane disruption from those that have a different MOA (Figure 2). In other words, since traditionally AMPs have been studied in the context of their ability to kill bacteria by disrupting bacterial membranes (Hancock and Sahl, 2006; Zasloff, 2009; Kumar et al., 2018; Lázár et al., 2018), most methods used to determine MOA focus first on determining whether the HDP is membrane perturbing. If it is not, very often, then and only then are other MOAs explored.

Rather than using a process of elimination, it is conceivable that future studies will rely on other parameters. As outlined in Haney et al. (2019), an increasing number of studies show that HDPs exert their antibacterial functions at least in part by targeting other cell components or through a variety of MOAs (Libardo et al., 2017). Instead of focusing solely on membrane damage, proteomic or transcriptomic profiling could be used since several HDPs have been observed to cause substantial changes in gene expression responses (Tomasinsig et al., 2004; Overhage et al., 2008; Zhu et al., 2013; Wenzel et al., 2014, 2015; Le et al., 2016; Wang et al., 2017). For example, Wenzel et al. (2012) used 2D-PAGE to investigate which proteins were up- or down-regulated in *Bacillus subtilis* upon exposure to a variety of lantibiotics. Select protein markers were identified to distinguish the impact of the tested lantibiotics on the cell envelope. Alternatively, Wuerth et al. (2019) examined the host response to *P. aeruginosa* in the absence and presence of the HDP IDR-1002 using RNA-Seq to provide insight into the MOA of 1002.

## Biophysical Techniques to Determine MOA

In addition to probing MOA in the presence of bacteria, a number of biophysical methods can be used to pinpoint HDP function. These typically rely on bacterial components (e.g., DNA from *S. aureus* to test whether an HDP targets DNA) or model systems (e.g., synthetic lipids or lipid extracts, nucleotides, etc.). Although these approaches are usually quite a bit more labor-intensive than the biological assays outlined above, they provide crucial information on the MOA at a molecular level. In particular, biophysical *in vitro* experiments provide important insights when the MOA does not involve membrane damage. In the following sections, we will briefly summarize some of the typical methods used.

### Membrane Disruption

There are many biophysical techniques to determine whether an HDP disrupts membrane integrity. Again, this is likely due to the fact that the membrane perturbation MOA (Figure 2) has been the focus of many studies over the years (Hancock and Sahl, 2006; Zasloff, 2009; Kumar et al., 2018; Lázár et al., 2018).

#### Pyranine Leakage Assay

Pyranine (8-hydroxy-1,3,6-pyrenetrisulfonate) is a pH sensitive fluorescent probe that can be used to detect proton concentration within lipid vesicles. It is a hydrophilic polyanionic molecule with an ionizable 8-hydroxyl group (pK_a_ = 7.2) dependent on the pH of the surrounding medium. The anionic character ensures no significant binding between pyranine and the negatively charged phospholipid vesicles, typically used as a model for the bacterial cytoplasmic membrane. Also, the fluorescence intensity is dependent on the extent of ionization in a pH 6–10 range (Clement and Gould, 1981). These characteristics enable the use of pyranine to detect proton and counterion transport across vesicle membranes in the presence of HDPs that function by causing membrane damage (Prabhananda and Kombrabail, 1996; Zhang et al., 2014). This assay can be viewed as a counterpart to the DiSC_3_5 assay described above. The additional information that can be probed using this assay is how the leakage depends on membrane composition or the presence of ions important for activity, e.g., Ca^2+^ (Zhang et al., 2016).

#### Calcein Leakage Assay

Damage induced to model membranes by HDPs, such as large unilamellar vesicles (LUVs) or small unilamellar vesicles (SUVs) can be studied using the calcein leakage assay. Calcein or carboxyfluorescein are fluorescent probes that are membrane impermeable and self-quench at high concentrations (Peisajovich and Shai, 2003; Tatulian and Kandel, 2019). Similar to the pyranine leakage assay, the dye is mixed with liposomes (e.g., using a number of freeze-thaw cycles) and the non-entrapped calcein is removed by using gel filtration. HDP-induced membrane damage leads to calcein leakage, and results in an increase in fluorescence (Peisajovich and Shai, 2003; Fillion et al., 2014). This assay can also be adapted for use in bacterial cells to test whether an HDP has an effect on bacterial cytoplasmic membrane integrity (Miyoshi et al., 2017). Finally, smaller fluorophores, such as the Ca^2+^-dependent fluorophore Quin-2 (Tatulian and Kandel, 2019), can be used to probe pores of a smaller diameter (<1 nm).

### Membrane Interaction

Since many HDPs function by translocating through the membrane (Park et al., 2009; Chileveru et al., 2015; Haney et al., 2019), methods to determine how HDPs interact with model membranes are important. One approach is to use oriented circular dichroism (OCD). In OCD, oriented lipid bilayers are used to provide information about the membrane alignment of peptides (Bürck et al., 2016). The observed signal allows a clear distinction to be made between a parallel versus perpendicular orientation of a peptide relative to the membrane bilayer (Bürck et al., 2016; Kumagai et al., 2019). Although this method can be applied to HDPs that adopt β-sheet conformations (Heller et al., 1998), most examples in the literature are for α-helical peptides (Lee et al., 2004; Pan et al., 2007; Cheng et al., 2011). Peptides that form clearly defined pores will display a change in the CD signal as a function of increasing peptide concentration. HDPs that translocate through the membrane do not display this change in CD signal (unpublished).

Alternatively, methods such as differential scanning calorimetry (DSC) (Arias et al., 2017; Jobin and Alves, 2019) and ^2^H or ^31^P solid-state NMR (Pan et al., 2007; Cheng et al., 2011; Fillion et al., 2014) can be used to determine whether HDPs affect lipid packing. HDPs that perturb the arrangement of fatty acyl chains result in changes in thermotropic-phase behavior as compared to lipid alone, as observed in the DSC thermogram. This can be used to indicate peptide integration, as demonstrated in a number of studies in the literature (Thennarasu et al., 2005; Pabst et al., 2008; Wenzel et al., 2014; Leber et al., 2018). HDPs that translocate through the membrane do not show any changes in the DSC thermogram (Wieczorek et al., 2010). Changes in ^31^P chemical shift depend on the change in orientation of the phosphorus nuclei in lipid membranes and provide additional information on HDP mode of action (Baila et al., 2004). By using phospholipids with deuterated acyl chains, ^2^H NMR can examine the effect of the HDP on acyl chain order (Marcotte et al., 2003). Recently, DSC has been used to probe the interaction of the AMP MSI-78 in whole bacteria (Brannan et al., 2015). Likewise, ^31^P NMR studies on whole bacterial cells have also been reported (Overall et al., 2019).

### DNA or RNA Interaction

The interaction of HDPs with DNA or RNA can be monitored using gel electrophoresis. The electrophoretic mobility of the DNA (or RNA) bands is typically examined as a function of HDP concentration. Binding between the HDP and DNA is defined as decrease in the band migration rate or a complete inhibition of band migration (Nam et al., 2014). The bands are stained with ethidium bromide, a fluorophore that can intercalate between base pairs of the DNA double helix and leads to a fluorescence increase, visualized under UV illumination (Cooper, 2011). For example, the DNA and RNA binding activity of buforin II, a potent HDP that causes rapid cell death of *Escherichia coli* without cell lysis, was determined using agarose gel electrophoresis (Park et al., 1998). Similarly, the DNA and RNA binding activity of 13-amino acid long indolicidin was studied. From the gel retardation experiments, it was found that indolicin, an HDP that also causes *E. coli* membrane permeabilization without cell lysis, binds DNA but not RNA (Hsu et al., 2005). Other examples are listed in Table 1.

### Nucleotide Interaction

Host defense peptides can interact with nucleotides such as ATP as part of their MOA (e.g., Hilpert et al., 2010). In biofilms, one important MOA is for HDPs to interact with the signaling nucleotide (p)ppGpp (Figure 3). This interaction can be examined by a co-precipitation assay, in which samples are mixed in microtiter plates and the increase in turbidity is quantified by an absorbance measurement at 620 nm. Using this assay, IDR-1018, a broad spectrum antibiofilm peptide, was found to preferentially bind ppGpp (de la Fuente-Núñez et al., 2014). The binding interaction leads to ppGpp degradation in bacterial cells and blocks the stress response, which consequently leads to biofilm prevention or eradication/dispersal of preformed biofilms. Alternatively, HDP/nucleotide interactions can be monitored using ^1^H NMR (Hilpert et al., 2010) or ^31^P NMR (de la Fuente-Núñez et al., 2014), by either using the signal of individual nucleotides in solution or whole bacteria.

### Other Methods

Alternate approaches involve examining other types of binding interactions, such as for example, the ability of HDPs to bind proteins or to coat surfaces to prevent biofilm formation (Segev-Zarko et al., 2015). In the first case, methods such as co-precipitation can be used. For instance, the ribosomal protein binding activity of Bac71-35, an HDP within the cathelicidin family, was determined by checking its co-sedimentation activity with purified ribosomes. Initially, *E. coli* 70S ribosomes were incubated with different concentrations of the peptide, and then the ribosome bound peptide was separated by ultracentrifugation. The ribosome pellets were analyzed by immunoblotting in order to confirm the presence of a Ba71-35/ribosomal protein interaction (Mardirossian et al., 2014). Alternatively, the presence of peptide on the surface of or within bacteria or on a solid support can be detected by labeling the peptide with rhodamine (Segev-Zarko et al., 2015) or other fluorescent dyes (Okuda et al., 2013), or by adding a His-tag (Ansari et al., 2017). In this case, it is important to verify that the label does not affect the structure and activity of the peptide. Finally, other types of binding interactions (e.g., with lipid II or LPS) can be examined using methods such as NMR, isothermal titration calorimetry (ITC) (e.g., thanatin, Sinha et al., 2017) or surface plasmon resonance (SPR) (e.g., Cg-Defh1, Schmitt et al., 2010; Table 1).

## Conclusion

A number of studies (Haney et al., 2015; Tucker et al., 2018; Yount et al., 2019) and the large number of HDP database entries (Fan et al., 2016; Pirtskhalava et al., 2016; Wang et al., 2016) clearly indicate that the HDP landscape is large and diverse (Haney et al., 2019). Not only are there many sequences, but HDPs can function using one or more MOAs (Figures 2, 3 and Table 1). To add to this complexity, some HDPs can have more than one function: antibacterial and antibiofilm, as considered in this review; or any combination of functions listed in Figure 1. Currently, HDP sequences are modified “by hand” or using more sophisticated library-based approaches [e.g., using SPOT synthesis (Winkler et al., 2009)] in order to optimize one function. However, the manner in which peptide sequence relates to specific functions or combinations of functions remains elusive. One way to improve our understanding of HDPs is to elucidate the mechanistic details underpinning the multiple functions of HDPs.

In this review, we examined the various tools at our disposal to probe the MOA of HDPs that function as antibacterial and/or antibiofilm agents. In a first step, it is crucial to have methods to determine the activity of a large set of HDPs. Methods to determine MICs, MBICs, and MBECs in a rapid and reliable manner are widely available. Next, assays and techniques to determine MOAs are applied. Depending on the MOA (Figures 2, 3), the choice of method (Table 1) can vary. For HDPs that function by perturbing the membrane for example, there are many biological and biophysical methods that provide complementary information. On the other hand, there are only a few select biophysical techniques to probe other MOAs. In this review, a number of examples that make use of biophysical methods to determine MOAs that involve DNA/RNA targets (Park et al., 1998; Hsu et al., 2005; Nam et al., 2014), protein targets (Mardirossian et al., 2014, 2018; Krizsan et al., 2015), stringent response inhibition (de la Fuente-Núñez et al., 2014; Pletzer et al., 2017b), and degradation of biofilms (Segev-Zarko et al., 2015; Ansari et al., 2017) were presented. A number of examples of how these biophysical approaches can be applied to bacteria (i.e., termed “biological” assays here) (de la Fuente-Núñez et al., 2014; Brannan et al., 2015; Miyoshi et al., 2017; Overall et al., 2019) serve to illustrate not only how the lines between these methods may become increasingly blurred in the future, but also how all the methods presented in this review, as well as direct visualization approaches [e.g., scanning electron microscopy (SEM) (Chileveru et al., 2015)] and other new approaches [e.g., small angle X-ray scattering (Von Gundlach et al., 2016, von Gundlach et al., 2019)], will continue to be relevant.

As it is clear from the examples given in Yasir et al. (2018), it is only in the last decade of the AMPs 40 year history that researchers have examined in detail how HDPs function as antibacterial and/or antibiofilm agents (i.e., Table 1 clearly shows that the assays/techniques listed have been more extensively used for the antibacterial MOA, as compared to the antibiofilm MOA). As more studies will report on the multifunctional, multi-MOA characteristics of HDPs, our understanding of the HDP landscape will improve. The details provided by the combination of sequence, function, and MOA data will allow us to rapidly zoom in to select areas of the HDP landscape to find effective drugs against diseases, including antimicrobial resistant ones. Many methods at our disposal are already high-throughput. A judicious combination of these methods and techniques that provide mechanistic details will enable us to find concrete solutions to the AMR problem in a timely manner.

## Author Contributions

NR and SS contributed to the writing of this review equally.

## Conflict of Interest

The authors declare that the research was conducted in the absence of any commercial or financial relationships that could be construed as a potential conflict of interest.

## Footnotes

1 http://aps.unmc.edu/AP/main.php

2 http://dbaasp.org
